# CO_2_ Adsorption Capacities in Zeolites and Layered Double Hydroxide Materials

**DOI:** 10.3389/fchem.2019.00551

**Published:** 2019-08-06

**Authors:** Cristina Megías-Sayago, Rogéria Bingre, Liang Huang, Gaëtan Lutzweiler, Qiang Wang, Benoît Louis

**Affiliations:** ^1^ICPEES – Institut de Chimie et Procédés pour l'Energie, l'Environnement et la Santé, Energy and Fuels for a Sustainable Environment Team, UMR 7515 CNRS – Université de Strasbourg – ECPM, Strasbourg, France; ^2^Environmental Functional Nanomaterials (EFN) Laboratory, College of Environmental Science and Engineering, Beijing Forestry University, Beijing, China; ^3^INSERM, UMR 1121, Strasbourg, France

**Keywords:** CO_2_ adsorption, zeolite, LDH, zeolite@ LDH, Si/Al, hierarchical porosity

## Abstract

The development of technologies that allow us to reduce CO_2_ emissions is mandatory in today's society. In this regard, we present herein a comparative study of CO_2_ adsorption over three types of materials: zeolites, layered double hydroxides (LDH), and zeolites coated LDH composites. The influence of the zeolite Si/Al ratio on zeolites sorption capacity along with the presence of mesopores was investigated. By comparing these results with the well-known performance of LDHs, we aim to provide insights on the factors that may influence the CO_2_ capture capacity over zeolites, thus providing useful tools for tuning their properties upon post-treatments.

## Introduction

The tremendous increase in the use of fossil fuels in the past 200 years has contributed to a steady rise of CO_2_ level in the atmosphere (Smil, 2017). Being the main actor responsible for the greenhouse effect, it led to reach a global warming never seen before, coming close to the pessimistic scenario (Stanley, 2003). Several efforts have been made to reduce those emissions, either by optimization of the current industrial processes, or by implementation of renewable energies where no CO_2_ is produced (Princiotta, 2011; Álvarez et al., 2017). However, the latter remain at a non-sufficiently mature state, hampered by high costs for their implementation and maintenance, mainly in developing countries (Mustapa et al., 2010; Horbach et al., 2014). Hence, this leads to the need to continue depleting fossil fuels to generate energy, and consequently, allow further release of CO_2_ in the atmosphere. Unfortunately, fossil fuels are not the unique factor increasing carbon footprint nowadays. A significant contribution comes from several industrial activities such as cement or steel production, whose CO_2_ emissions dramatically contribute to accelerate climate change.

CO_2_ capture technologies have been recently implemented in industries. However, the sole commercially mature technology to capture CO_2_ remains an amine-based scrubbing, but its high cost and hazardous by-products forces mankind to find out alternative technologies (Veltman et al., 2010; Vericella et al., 2015; Chen C. et al., 2017).

The development of new solid adsorbents / sorbents has flourished in the last decade, being preceded by Sircar's group work (Sircar et al., 1996). The latter sorbents can be classified into low-temperature (< 200°C), intermediate-temperature (200–400°C), and high-temperature (> 400°C) CO_2_ adsorbents/sorbents depending on their working temperature range (Wang et al., 2012, 2015; Cao et al., 2014). LDH, known as hydrotalcite-like compounds, are a class of layered materials which comprise mono- or di- and trivalent cations. To date, several studies have been dealing with the high potential of LDHs-derived CO_2_ adsorbents, especially conventional Mg-Al LDHs, which demonstrated a great performance at high temperatures (Alpay and Ding, 2000; Hutson and Attwood, 2008; Sharma et al., 2008; Lwin and Abdullah, 2009; Chang et al., 2013). Likewise, zeolites have also been subjected to extensive research for the adsorption of CO_2_ due to their high capture capacity, low regeneration temperatures and high stability over several adsorption-desorption cycles (Siriwardane et al., 2005; Pham et al., 2014; Bande et al., 2015; Chen C. et al., 2017; Hasegawa and Matsumoto, 2017). Regarding sorbents working at intermediate temperature range, recent studies have shown a great potential from LDH coated zeolites (Li et al., 2018). A considerable increase in the adsorption of CO_2_ was observed after the coating of LDH over a zeolite core at temperatures ranging from 30 to 300°C (Othman et al., 2006).

Herein, we present a CO_2_ adsorption study comprising the three types of aforementioned materials. The impact of the zeolite Si/Al ratio on its capture capacity, as well as the presence of mesopores has been highlighted. By comparing these results with the well-known performance of LDHs and LDH@zeolite composites, we aim to give insights on the factors that may influence the CO_2_ capture in zeolites, thus providing useful tools to tune their features upon different post-treatments (LDH coating, introduction of mesoporosity).

## Experimental

### Synthesis

#### Large Crystal Zeolites

NH_4_-ZSM-5 zeolites were directly synthesized via fluoride-mediated route adapted from our former studies. In a typical synthesis, the Al-source (different amounts to reach the following Si/Al molar ratios: 200, 100, 50, and 25) was first weighted in a 150 mL-Erlenmeyer flask where 50 mL of distilled water was added under vigorous stirring (700 rpm, r.t.). After complete dissolution, TPABr (0.498 g) and NH_4_F (1.103 g) were consecutively added. Finally, 1.607 g of solid silica (Aeroperl 300/30, Evonik) was slowly added during 5 min. The gel was aged 2h under vigorous stirring and finally autoclaved at 443 K for 144 h. H-zeolite form was easily obtained after a single calcination at 823 K during 15 h in static air, needed to completely remove the template. The samples were named as H-ZSM-5-A, H-ZSM-5-B, H-ZSM-5-C and H-ZSM-5-D, respectively, following the order related with the Si/Al molar ratio (usually denoted as SAR) decrease.

#### Zeolite Derived Materials

Commercial ZSM-5 zeolite (CBV3020E) having Si/Al molar ratio of 15 was purchased from Zeolyst in its ammonium form. The zeolite was either calcined at 600°C for 4 h to produce its proton form, named R0-H, or subjected to a cationic exchange to produce its sodium form R0-Na. The latter was performed by exchanging three times the zeolite with NaCl 1 M at 80°C for 1h, followed by filtration and drying. To prepare the mesoporous zeolite, 200 mg of the commercial powder in its proton form was subjected to an alkaline treatment with 0.15 mL of 0.2 M of NaOH at 65°C for 30 min. The solution was filtered, dried and added to a 30 mL solution of tetraethylammonium hydroxide (TEAOH, 0.1 M) and 100 mg of alkali lignin (Aldrich). After stirring, the solution was transferred to an autoclave and placed in an oven at 150°C for 10 h, followed by filtration, drying and calcination at 550°C for 15 h (1 h ramp to burn off the templates). The sample was treated three times with 1 M of NH_4_Cl at 80°C, followed by filtration, drying and calcination at 550°C to release NH_3_ to ensure the proton form of the zeolite. The sample was named R0-meso-H. Following the same procedure as for R0-H, the mesoporous sample was subjected to another cationic exchange to leave its sodium form R0-meso-Na.

R0-H material was also coated with Mg-Al LDH using a co-precipitation method. It should be noted that based on the obtained results (H-ZSM-5-D vs. R0-H), only the commercial sample was chosen for the coating procedure. Firstly, 200 mg of zeolite was dispersed in 40 mL of distilled water for 30 min, followed by the addition of 0.212 g of sodium carbonate and dispersion for additional 20 min. Then, a 40 mL aqueous solution containing 1.918 mmol of magnesium nitrate hexahydrate (Mg(NO_3_)_2_.6H_2_O) and 0.955 mmol of aluminium nitrate nonahydrate (Al(NO_3_)_3_.9H_2_O) was added drop-wise to the previous solution for around 1 h under vigorous stirring. The pH of the solution was kept constant at 10 by adding 1 M of sodium hydroxide. After stirring for 4 h, the solid was filtered and washed. The sample was stirred for another 4 h in 40 mL of ethanol, and then filtered and dried. The composite was calcined at 400°C for 5 h to obtain the LDO (layered double oxide) and named finally R0@LDH.

#### LDHs

MgAl and CaAl LDHs were prepared using the co-precipitation method. All chemicals were purchased from Acros Organics, VWR and Sigma-Aldrich. In brief, a salt solution containing a mixture of M^2+^ precursor and Al(NO_3_)_3_ 9H_2_O was added dropwise to an alkaline solution (100 mL) containing Na_2_CO_3_ (NaNO_3_ for CaAl LDHs) and additives. The pH of the precipitation solution was kept constant at 10 (or 11 for CaAl LDHs) by addition of NaOH (4M) solution. The resulting mixture was aged at room temperature for 12 h under continuous stirring. The aged mixture was then filtered and washed with deionized water followed by drying at 100°C in an oven. In order to study the impact of structural degradation (LDO formation), MgAl LDH sample was finally calcined at 400°C during 5 h in static air. Meanwhile, CaAl LDH was treated at 750°C during 5 h in the same atmosphere. Samples were named as LDH fresh or LDH calcined.

### Characterization

Powder X-ray diffraction (XRD) patterns of zeolites were acquired using a Bruker D8 (Cu Kα) diffractometer operated at 40 kV and 40 mA. XRD patterns were recorded in the 2θ = 5–60° range with a step size of 0.05°. XRD patterns of LDH materials were acquired over a Rigaku Ultima IV apparatus using CuKα radiation (λ = 0.15418 nm), operated at 40 kV and 20 mA. Scans were performed at 2° min^−1^ in the 3–70° range.

SEM images were acquired in a JEOL FEG-6700F microscope working at a 9 kV accelerating voltage. Bulk elemental analyses were determined by X-ray fluorescence (XRF) using a XEPOS (AMETEK) device equipped with a Rh radiation tube.

The textural properties were analyzed by means of nitrogen physisorption using Micromeritics ASAP 2420 equipment. Samples were pre-treated *in-situ* at 200°C under vacuum prior to the analyses. Surface areas were determined using the BET method and pore size distributions were obtained using the adsorption branch of the isotherms (BJH method).

Zeolite acid site density was evaluated by temperature programmed ammonia desorption (NH_3_-TPD) by using a Micromeritics AutoChem II chemisorption analyzer equipped with TCD detector. Samples were pre-treated *in-situ* at 550°C during 1 h under helium atmosphere. After that, samples were cooled down to room temperature and a 5% NH_3_/He flow was passed through the reactor. Once physisorbed ammonia was removed, the temperature gradually increased to 550°C.

The hydrophilicity of the zeolites has been determined by contact angle measurements as follows: 20 mg of powder was placed under an hydraulic press, and let under 12 bar for 30 s to obtain a compact pellet. Wetting properties of the samples were determined by static contact angle using an optical tensiometer (Attension theta, Biolin Scientific). A 2 μl drop of purified water (milli Q) was deposited on the pellet surface and the angle was recorded by the camera mounted on the device.

### Evaluation of CO_2_ Adsorption Capacity

Thermogravimetric sorption of CO_2_ on zeolites, LDHs and composites was measured using a TGA analyzer (Q500 TA Instrument). All solids were first calcined in a muffle furnace at a temperature comprised between 400 and 600°C for 5 h, before transferring the samples to the TGA analyzer. To avoid any error, the test was done immediately after the first calcination. Finally, the samples were further calcined *in situ* for 1 h in N_2_ prior to the measurement. CO_2_ adsorption experiments were carried out at 1 atm with a constant flow of CO_2_ (40 mL min^−1^).

## Results and Discussion

### Large Zeolite Crystals

Fluoride-mediated synthesis of ZSM-5 zeolite crystals provides mild conditions, thus facilitating the formation of few nuclei under diluted conditions as consequence of a slow release of SiO_x_${\text{F}}_{\text{y}}^{-}$ species (Flanigen et al., 1986; Losch et al., 2017). The latter renders possible the formation of highly crystalline zeolites characterized by larger crystals (usually tenths of microns) with less defects and more open structures than in alkaline medium (Xu et al., 1990). This, together with the high Si/Al ratio that can be obtained, entails the formation of more hydrophobic materials. Therefore, the possible effect of both the hydrophobicity and crystal size can be evaluated in terms of CO_2_ capture capacity.

**Figure 1:**
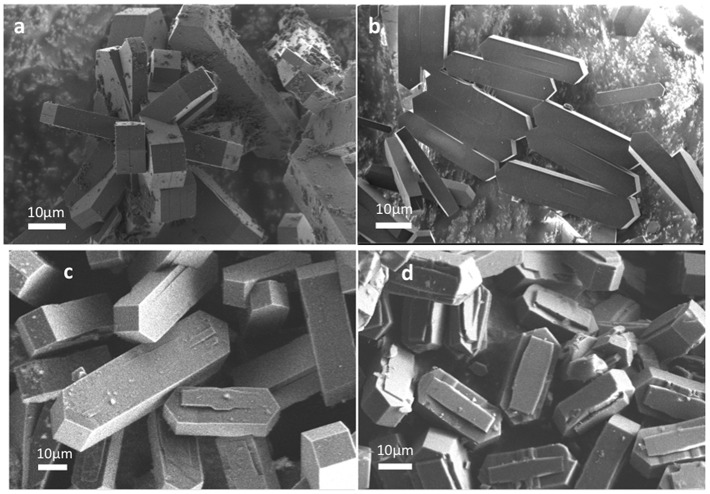
SEM images of as-synthetized H-ZSM-5 zeolites. **(a)** H-ZSM-5-A, **(b)** H-ZSM-5-B, **(c)** H-ZSM-5-C, and **(d)** H-ZSM-5-D.

The XRD patterns of each zeolite (Figure S1) confirmed the presence of the sole MFI structure (Ocampo et al., 2009, 2010). Whatever the aluminum content in the framework, all syntheses led to the formation of large prismatic crystals (Figure 1) having between 30 and 60 μm in size. Lower Al containing zeolites have, in general, narrow crystal size distributions (Figures 1a,b) differing from zeolites with higher Al content (Figure 1c). A difference in the crystal morphology can be observed for H-ZSM-5-D (possessing the lowest SAR, Table 1).

**Table 1:** Chemical composition, acid site density and textural properties of as-synthetized H-ZSM-5 zeolites.

**Sample**	**SAR (XRF)**	**Acid site density (mmol(H^**+**^)/g zeolite)^a^**	**S_**BET**_ [m^**2**^/g]**	**V_**pore**_ (cm^**3**^/g)**
H-ZSM-5-A	226	0.110	355	0.178
H-ZSM-5-B	115	0.168	296	0.184
H-ZSM-5-C	55	0.297	348	0.178
H-ZSM-5-D	43	0.309	347	0.181

a *Determined by NH_3_-TPD*.

Table 1 summarizes the Si/Al ratios, the total number of Brønsted acid sites and the textural properties of as-prepared zeolites. XRF values together with NH_3_-TPD experiments confirm a successful variation of acidic properties, i.e., aluminum content, within the series while neither influencing the specific surface areas, nor the pore volumes.

**Figure 2:**
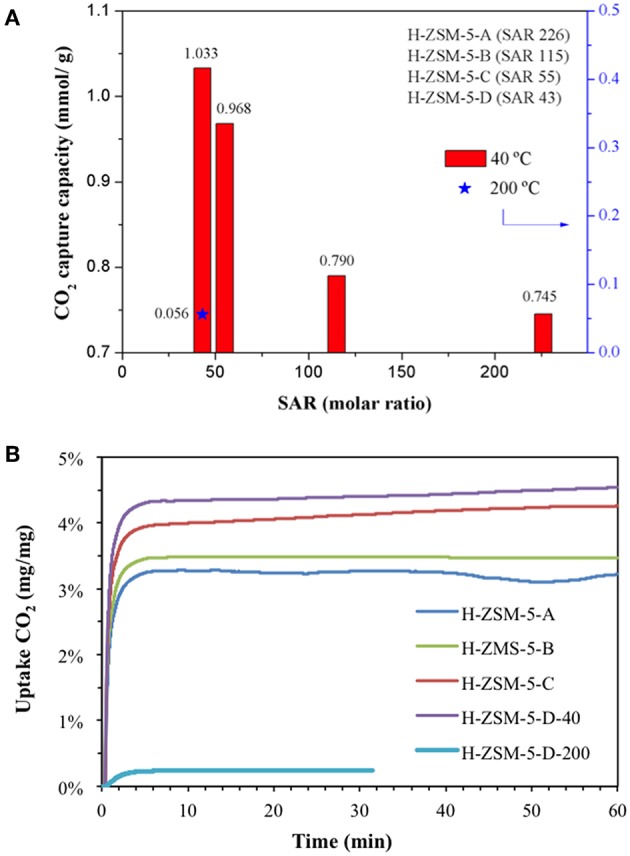
**(A)** CO_2_ capture capacity as a function of Si/Al mole ratio and sorption temperature. **(B)** CO_2_ uptake curves.

CO_2_ capture capacities of large crystals H-ZSM-5 zeolites were evaluated using a TGA analyzer in order to monitor the weight changes of each sample under a constant CO_2_ flow. Prior to analysis, the sample surface was cleaned by means of two calcination steps *in situ* under inert flow. Afterwards, CO_2_ capture abilities were evaluated at low temperature (40°C) and intermediate-temperature (200°C) and presented in Figure 2. The data acquired at low temperature strongly depend on the Si/Al content in zeolites (Figure 2A) which is in agreement with former literature reports (Chen H. et al., 2017). CO_2_ adsorption capacity was enhanced while diminishing the Si/Al ratio. This can be attributed to the presence of more Al-atoms in the framework. CO_2_ interaction with zeolites is governed by the hard Lewis base character of oxygen atoms (Pham et al., 2014) that strongly interact with hard Lewis acid sites as Al^3+^. One may therefore *a priori* expect that lower Si/Al ratio may induce a higher quantity of CO_2_ adsorbed. Indeed this trend could be verified over large MFI zeolite crystals. In addition, lower Si/Al implies superior ion exchange ability due to the presence of more charge compensation cations. Finally, zeolites with lower Si/Al ratio are expected to be more hydrophilic. Indeed, contact angle measurements have been performed and hopefully confirmed this statement. H-ZSM-5-D, having the lowest SAR (43), exhibited the smaller contact angle with the water drop, 22.6° in comparison with 28.5° for H-ZSM-5-C (SAR 55), 55.0° for H-ZSM-5-B (SAR 115) and 67.0° for H-ZSM-5-A (SAR 226), respectively.

To summarize, the higher CO_2_ sorption capacity has been obtained over zeolites which are more hydrophilic, thus possessing more Al-atoms in the frame.

The adsorption capacity at intermediate-temperature (200°C) was also evaluated over H-ZSM-5-D (lowest SAR zeolite) and presented in Figure 2A. The adsorption capacity diminished from 1.033 to 0.056 mmol_CO2_/g, limiting the application of large ZSM-5 crystals at higher temperatures. The latter result points out the importance of physisorption phenomena in zeolites at low temperatures. In contrast at higher temperatures, CO_2_ sorption should be only ruled by chemical interactions, which unfortunately diminishes the sorption capacity.

CO_2_ uptake curves as function of time are shown in Figure 2B. The sorbents displayed fast CO_2_ sorption kinetics for a very short absorption time period (less than 3 min). The latter could be attributed to the high specific surface area and the microporous network which provide a higher availability of active sites. In general, two stages concerning the sorption process can be differentiated in the solids. Firstly, a fast uptake reaction occurs at the initial stage of CO_2_ absorption (less than 3 min). Secondly, a later long-period stage usually appears where it is possible to distinguish a continuous (but considerably slower) increase in the sample weight. The first stage is directly related to the chemical reaction control whilst the second one is more likely attributed to a diffusional control (Chang et al., 2013). By careful comparing these uptake curves obtained at 40°C (Figure 2B), it is noteworthy that the sorption process is controlled by the chemical reaction for low Al content H-ZSM-5-A (SAR 226) and H-ZSM-5-B (SAR 115) zeolites. In contrast, H-ZSM-5-C (SAR 55) and H-ZSM-5-D (SAR 43) seem to suffer from diffusional limitations. These results are in line with the adsorption capacity values presented in Figure 2A. As aluminum content increases in the materials, a larger amount of CO_2_ molecules diffuse and absorb within the pores, thus hindering the CO_2_ diffusion along the cavities. This phenomenon is discarded at 200°C due to the lower uptake capacity at higher temperatures.

### Core-Shell LDH@zeolite Composites

**Figure 3:**
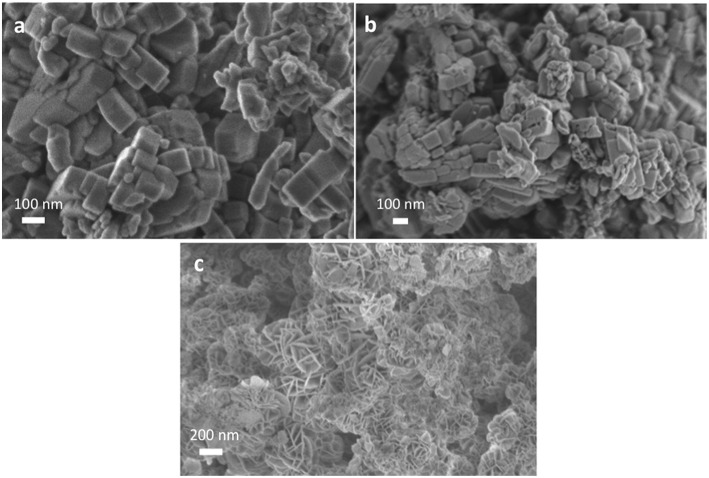
SEM pictures of **(a)** R0-H, **(b)** R0-meso H, and **(c)** R0@LDH composite.

The diffraction patterns of commercial R0-H zeolite and core-shell LDH@zeolite material confirm the sole presence of MFI structure (Figure S2). Neither the presence of mesopores nor the coating of LDH led to different diffraction peaks. The LDH presence is evidenced by SEM analysis as shown in Figure 3. R0-H sample possess regular nanosized morphology (Figure 3a) with smooth surface which became rough when the mesopores were created (Figure 3b). After coating with LDH, the composite retains the original shape of pristine zeolite together with uniform flower-like shaped edges (Figure 3c). A core-shell LDH@ZSM-5 zeolite composite has therefore been successfully obtained, as already reported by Wang et al. (Li et al., 2018).

The textural properties of commercial R0-H and post-modified materials are summarized in Table 2. The specific surface area of both zeolites (commercial and meso) is close to 360–370 m^2^/g, not being affected by the presence of mesopores. A slight decrease of about 20% in BET surface could be observed after the coating with LDH, R0@LDH sample, whilst the total pore volume almost doubled.

**Table 2:** Textural properties of commercial R0-H and zeolite derived materials.

**Sample**	**S_**BET**_ [m^**2**^/g]**	**S_**meso**_ [m^**2**^/g]**	**V_**total**_ (cm^**3**^/g)**	**V_**micro**_ (cm^**3**^/g)**
R0-H	369	147	0.23	0.109
R0-meso H	362	144	0.26	0.107
R0@LDH	298	142	0.43	0.076

**Figure 4:**
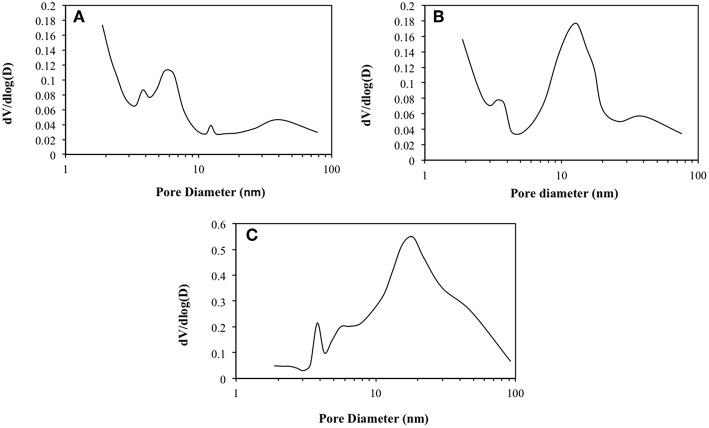
BJH pore profiles of **(A)** R0-H, **(B)** R0-meso H, and **(C)** R0@LDH.

In order to gain more insights on those samples porosity, BJH pore profiles are shown in Figure 4. The pore size variation toward the mesoporous range among the samples is verified while ranging from R0-H to R0-meso H and R0@LDH. Pristine zeolite is characterized by the sole presence of micropores (Figure 4A) together with mesopores having 4–6 nm being assigned to intercrystalline porosity. The pore diameter profile changes for R0-meso H (Figure 4B) highlighting the presence of mesopores of about 12 nm, demonstrating the mesoporous character of this sample. Regarding the zeolite coated with LDH (Figure 4C), the pore sizes further increased in the mesoporous range. The presence of macropores could also be evidenced, being probably due to the presence of LDHs at the zeolite crystals surface (Li et al., 2018). Zeolite micropores might be still accessible during nitrogen adsorption after the LDH coating procedure but the diffusion might be partially hindered. In any case, the use of zeolite for the synthesis of the LDH@ZSM-5 nanocomposite allows enhancing the specific surface area in comparison to pristine LDH.

**Figure 5:**
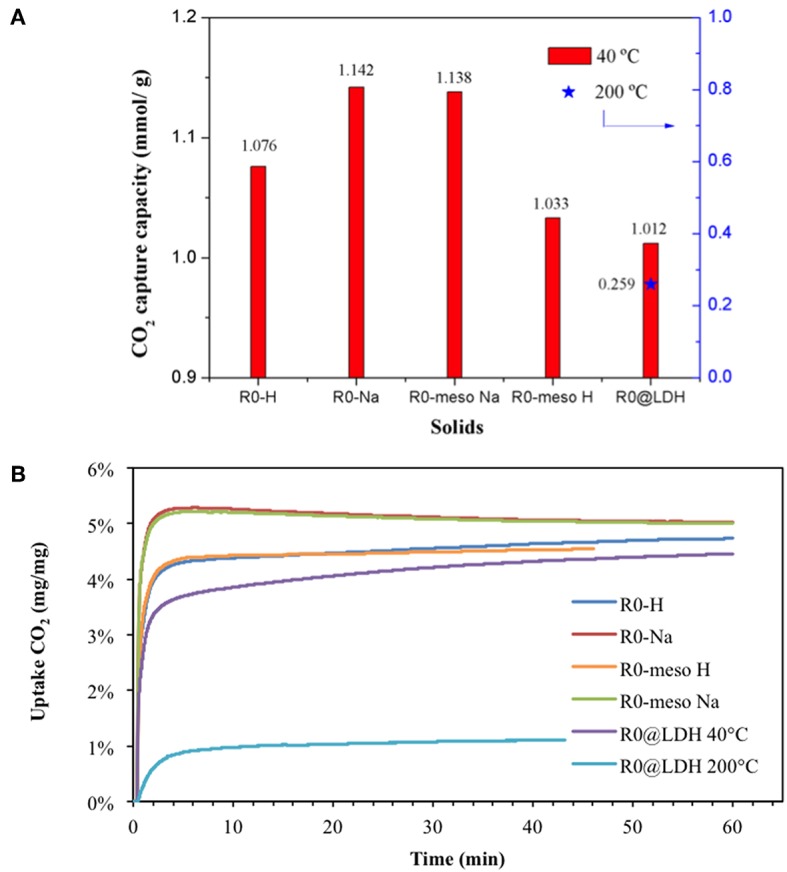
**(A)** CO_2_ capture capacity of zeolites and LDH-based materials as function of sorption temperature. **(B)** CO_2_ uptake curves.

CO_2_ capture capacities of as-prepared samples are presented in Figure 5A. In order to evaluate the cation nature influence along with the mesopore presence, R0-H and R0 meso-H samples were subjected to cationic exchange to get their sodium form. In principle, the CO_2_ sorption capacity raises when the protons are exchanged with sodium cations (R0-H vs. R0-Na and R0 meso-H vs. R0 meso-Na, respectively). Although some differences could be observed due to mesopores presence, a higher capacity could be achieved over Na-zeolite forms. Previous studies have underlined that the main interaction between the zeolite surface and CO_2_ is the field gradient—quadrupole interaction (Hasegawa and Matsumoto, 2017) which is proportional to *r*^−3^ and the valence of the cation, *z*. Since the ionic radius of H^+^ is the smallest, the ion-CO_2_ distance is the shortest and therefore the interaction would be the strongest. Carbon dioxide molecules in small and medium-pore zeolites are strongly adsorbed, possibly hindering the diffusion of other molecules inside the pores, resulting in smaller CO_2_ uptake capacities. The latter explains the lower observed uptakes for acidic R0 and R0-meso samples in comparison to their Na-exchanged counterparts.

The presence of mesopores (R0-H vs. R0-meso H) led to lower sorption capacities, in contrast to what could be expected taking into account mass transfer limitations. A possible explanation could be based on the removal of Al-atoms during the creation of the mesopores, diminishing therefore the number of potential adsorption sites for carbon dioxide molecules. Indeed, the sorption capacity achieved over R0-meso H sample is 1.033 mmolCO_2_/g, being exactly the same value reached over H-ZSM-5-D. The later zeolite has a Si/Al = 43, which is almost the triple than for R0-H (Si/Al = 15). This effect is partially suppressed after exchanging the zeolite with Na^+^, probably due to a better diffusion of carbon dioxide molecules through the narrow zig-zag channels, hence enabling an easier access to the active sites.

When the acidic R0 zeolite is coated with LDH, a lower sorption capacity is obtained. As observed in the BJH profiles, zeolite micropores seem to be blocked (at least partially) after LDH coating, suggesting that only the LDH part could interact with CO_2_. Although LDHs have demonstrated to be efficient CO_2_ adsorbents (Chang et al., 2013), the quantity used in this composite is smaller when compared to bare LDH material. At higher temperature (200°C), the capacity value diminishes from 1.012 to 0.259 mmol_CO2_/g (which supposes 76% capacity loss), as expected by the exclusion of physisorption phenomenon at higher temperatures. However, this decrement is low in comparison with the 95% registered for H-ZSM-5-D zeolite (previous section) which points out the beneficial effect of LDH coating.

Figure 5B shows the CO_2_ uptake curves as a function of time for each sample. It is worthy to mention that Na-exchanged samples and acidic zeolites follow the similar trend, being different from LDH coated sample. All samples are characterized by a fast adsorption process (less than 3 min) followed by a diffusional control effect depending on the material. Slight differences can be observed over micro- and mesoporous H-zeolites (R0-H vs. R0-meso H) regarding the second adsorption stage, i.e., the later long-period stage. For R0-H, it is possible to distinguish a continuous but considerably slower weight increase contrarily to R0-meso H sample. This effect suggests that the mesopores presence may avoid diffusional problems during the adsorption process. Likewise for Na-exchanged samples, a continuous decrease in the sample weight is observed, thus indicating that a small fraction of CO_2_ molecules are fastly and weakly adsorbed on the zeolite surface. The impact of diffusion is more pronounced over R0@LDH at 40°C, being limited at 200°C.

### LDHs

The diffraction patterns of MgAl and CaAl based samples are presented in Figure S3. Highly crystalline Mg-Al-NO_3_ and Ca-Al-NO_3_ were successfully prepared using co-precipitation method leading to pure LDHs phases (Wang et al., 2015) with characteristic peaks at 11.7°, 23.7°, 34.7°, and 39.3°, corresponding to the basal planes of (003), (006), (009) and (015) reflections (Climent et al., 2004). Since LDH derived materials will be activated by thermal treatment before CO_2_ sorption, XRD patterns were also recorded after calcination. LDHs usually lose their layered structure, turning into layered double oxides (LDOs) which possess three kinds of active sites. The structure and composition changes are probably the most important parameters to take into account for an application as sorbent. It can be deduced from TGA curves (Figure S4), that structural changes occur at different temperatures for MgAl and CaAl LDHs. MgAl LDH first loses its interlayer water until 200°C. From this temperature, the dehydroxylation of the octahedral layers takes place as well as the interlayer charge compensation nitrate anions removal, which last up to 400°C. The same two weight loss steps can be differentiated for CaAl LDH, being in this case 550°C the final temperature. Based on that and in order to ensure LDOs formation, MgAl and CaAl LDHs were calcined at 400°C and 750°C during 5 h, respectively, and XRD diffraction patterns presented in Figure S3. As expected, after calcination, the samples lost their original structure and changed to pure mixed-metal oxide phases, MgAl_2_O_4_ and CaAl_2_O_4_, respectively.

**Figure 6:**
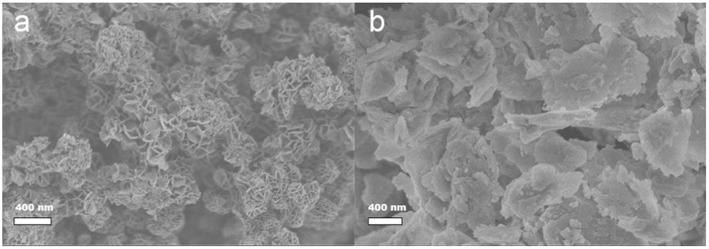
SEM images of **(a)** MgAl LDH and **(b)** CaAl LDH.

SEM images of LDH samples are presented in Figure 6. MgAl LDH exhibits a typical spheroidal rose-like morphology different from CaAl LDH where only regular aggregates could be observed.

The textural properties of fresh (LDH) and calcined (LDOs) solids are presented in Table 3. MgAl LDH specific surface area is more than twice higher than CaAl one. In both cases, the S_BET_ increased after calcination, although the increment is much higher for MgAl, reaching 224 m^2^/g. The pore sizes are similar for both samples, increasing for MgAl after calcination. Likewise to the pore volume, important differences between the samples could be detected, MgAl exhibiting a much higher pore volume. In both cases, the values increased after calcination, becoming more important for MgAl sample.

**Table 3:** Textural properties of fresh and calcined LDHs.

**Sample**	**S_**BET**_ [m^**2**^/g] fresh**	**S_**BET**_ [m^**2**^/g] calcined**	**Pore size (nm) fresh**	**Pore size (nm) calcined**	**V_**pore**_ (cm^**3**^/g) Fresh**	**V_**pore**_ (cm^**3**^/g) calcined**
MgAl LDH	50	224	9.0	13.3	0.19	0.63
CaAl LDH	21	26	10.4	10.0	0.04	0.06

**Figure 7:**
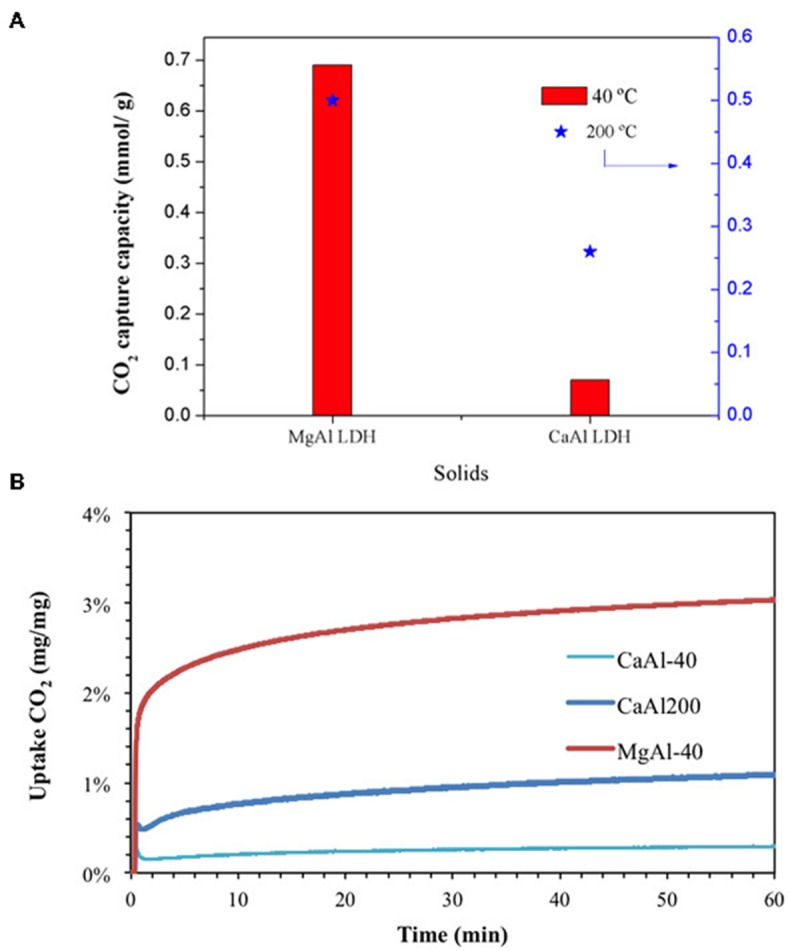
**(A)** CO_2_ capture capacity of calcined LDH as function of sorption temperature **(B)** CO_2_ uptake curves.

Carbon dioxide capacity values are presented for both samples in Figure 7A. The superiority of MgAl sample is evidenced at both temperatures, which seems logical taking into account the higher specific surface area and pore volume. Likewise for the uptake curves (Figure 7B), both samples are characterized by a fast adsorption (superior to the zeolites) and diffusional impediment in a second stage, being evidenced by a continuous weight increase as a function of time.

### Comparison of the Sorbents

In general, CO_2_ sorption capacity depends on different factors as discussed throughout this contribution. A careful control of these parameters for different materials is an effective tool to select the suitable application for the right material.

**Figure 8:**
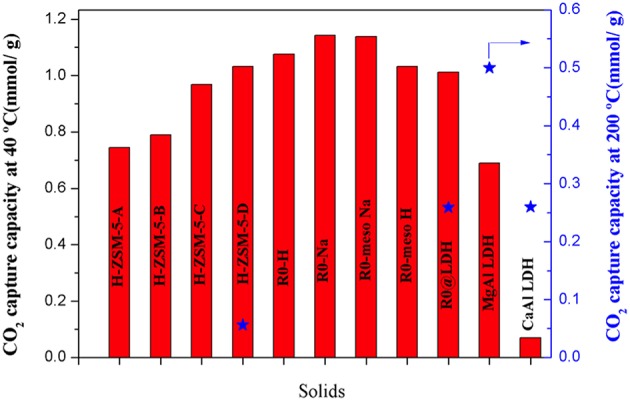
CO_2_ capture capacities comparison at different temperatures.

In order to summarize and properly compare, CO_2_ capture capacities of all materials are presented in Figure 8. At low temperature, the highest sorption values are registered for Na-exchanged zeolites. As aforementioned, the presence of mesopores did not have an important impact, contrarily to the Si/Al ratio and the cation nature. The impact of Si/Al ratio has been demonstrated for the large zeolite crystals (H-ZSM-5-A to H-ZSM-5-D samples). Likewise, the cation nature effect has been evidenced whatever the crystal size. While raising the temperature, the superior performance of LDHs is clear. Although at 40°C, R0@LDH's capacity remained lower than pristine zeolite (R0-H and R0-Na), at higher temperature the LDH coating led to a more efficient sorbent material, comparable to CaAl LDH. Nevertheless, the best potential sorbent at intermediate temperature remains MgAl LDH.

## Conclusion

The synthesis and characterization of large ZSM-5 zeolite crystals and LDH derived materials have been successfully performed. Those materials demonstrated efficient CO_2_ sorption capacity. It has been clearly shown that CO_2_ capture strongly depends on the concentration of Al-atoms in the zeolite framework as well as its cation nature. In contrast, the mesoporous character did not seem to induce an important effect.

For LDHs, high specific surface area combined with high pore volume appear as a determining factor for achieving high adsorption. When comparing different materials, the highest sorption values at low temperature were registered for sodium-exchanged zeolites. When enhancing the temperature up to 200°C, the superiority of LDHs has been clearly evidenced.

## Data Availability

All datasets generated for this study are included in the manuscript and/or the Supplementary Files.

## Author Contributions

All authors listed have made a substantial, direct and intellectual contribution to the work, and approved it for publication.

### Conflict of Interest Statement

The authors declare that the research was conducted in the absence of any commercial or financial relationships that could be construed as a potential conflict of interest.
