# Supplementary material for: CO2 Adsorption Capacities in Zeolites and Layered Double Hydroxide Materials
Source: Front Chem. 2019 Aug 6;7:551. doi: 10.3389/fchem.2019.00551 (PMC6691059; doi:10.3389/fchem.2019.00551)
Supplement: Supplementary file 1 [file Data_Sheet_1.pdf]

Supplementary material

CO_2 adsorption capacities in zeolites and layered double hydroxide materials

Cristina Megías-Sayago[1,*], Rogéria Bingre[1], Liang Huang[1,2], Gaëtan Lutzweiler[3], Qiang Wang[2], Benoît Louis[1]

[1]Institute of Chemistry, UMR 7177, University of Strasbourg, 1 rue Blaise Pascal F-67000 Strasbourg Cedex, France

[2]Environmental Functional Nanomaterials (EFN) Laboratory, College of Environmental Science and Engineering, Beijing Forestry University, Beijing 100083, China

[3] INSERM, UMR 1121, 11 rue Humann, 67085, Strasbourg, France

*** Correspondence:**

Cristina Megías-Sayago

megiassayago@unistra.fr

Figure caption

Figure S1. XRD patterns of as-prepared zeolites.

Figure S2. XRD patterns of commercial R0-H and derived zeolites.

Figure S3. A) X-ray diffractograms of A) MgAl and B) CaAl based samples

Figure S4. TGA profiles of MgAl LDH and CaAl LDH under N_2.

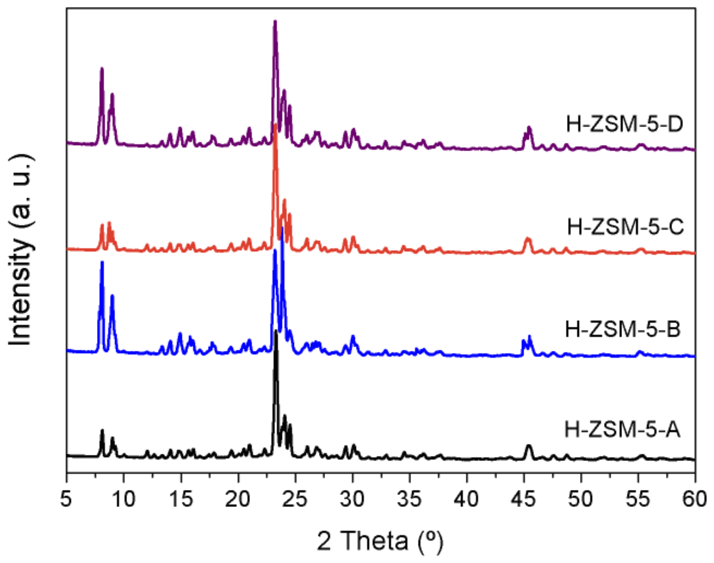

Figure S1. XRD patterns of as-prepared zeolites.

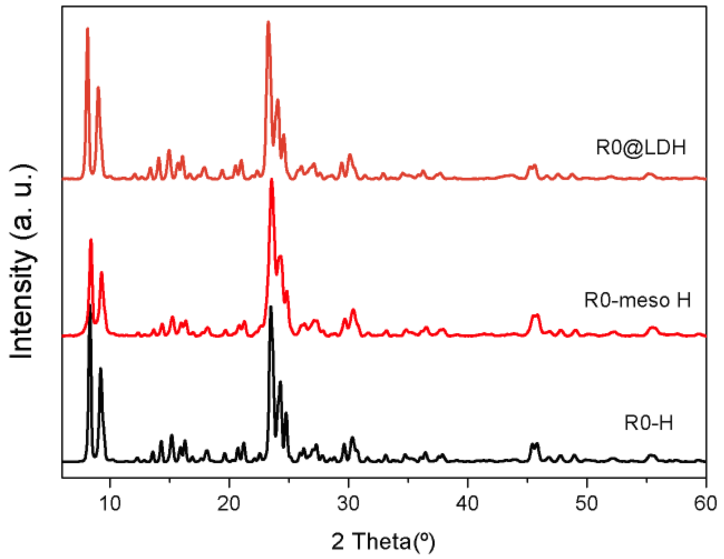

Figure S2. XRD patterns of commercial R0-H and derived zeolites.

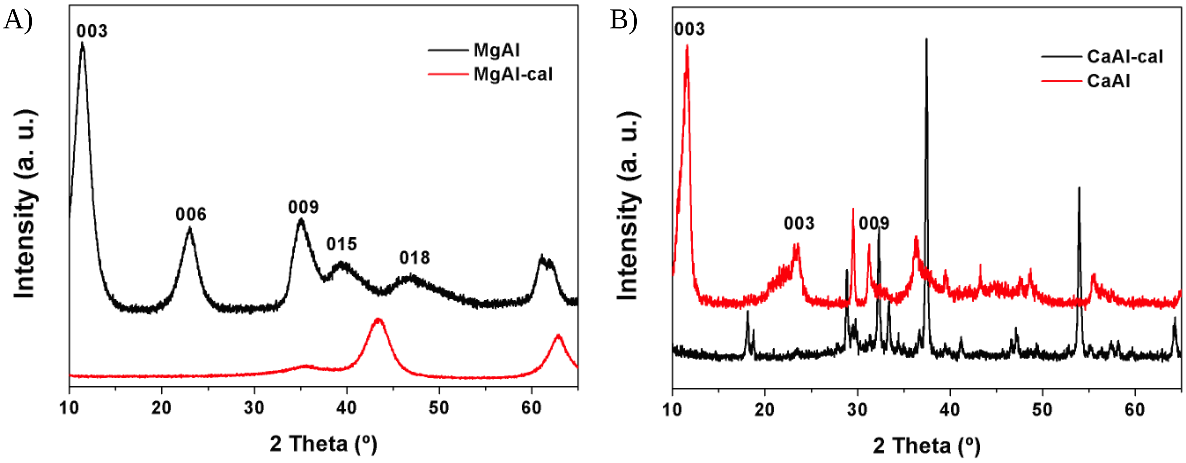

Figure S3. A) X-ray diffractograms of A) MgAl and B) CaAl based samples

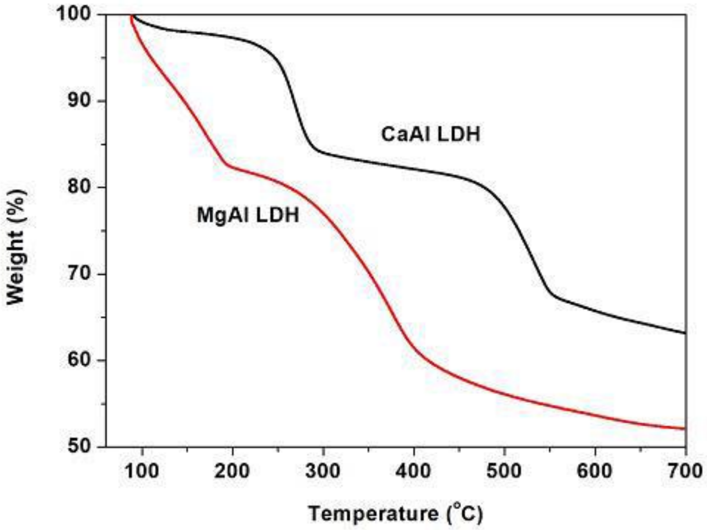

Figure S4. TGA profiles of MgAl LDH and CaAl LDH under N_2.
